# Exploiting the geometry of the solution space to reduce sensitivity to neuromotor noise

**DOI:** 10.1371/journal.pcbi.1006013

**Published:** 2018-02-20

**Authors:** Zhaoran Zhang, Dena Guo, Meghan E. Huber, Se-Woong Park, Dagmar Sternad

**Affiliations:** 1 Department of Bioengineering, Northeastern University, Boston, Massachusetts, United States of America; 2 Department of Physics, Northeastern University, Boston, Massachusetts, United States of America; 3 Department of Biology, Northeastern University, Boston, Massachusetts, United States of America; 4 Department of Mechanical Engineering, Massachusetts Institute of Technology, Cambridge, Massachusetts, United States of America; 5 Department of Electrical and Computer Engineering, Northeastern University, Boston, Massachusetts, United States of America; Northwestern University, UNITED STATES

## Abstract

Throwing is a uniquely human skill that requires a high degree of coordination to successfully hit a target. Timing of ball release appears crucial as previous studies report required timing accuracies as short as 1-2ms, which however appear physiologically challenging. This study mathematically and experimentally demonstrates that humans can overcome these seemingly stringent timing requirements by shaping their hand trajectories to create extended timing windows, where ball releases achieve target hits despite temporal imprecision. Subjects practiced four task variations in a virtual environment, each with a distinct geometry of the solution space and different demands for timing. Model-based analyses of arm trajectories revealed that subjects first decreased timing error, followed by lengthening timing windows in their hand trajectories. This pattern was invariant across solution spaces, except for a control case. Hence, the exquisite skill that humans evolved for throwing is achieved by developing strategies that are less sensitive to temporal variability arising from neuromotor noise. This analysis also provides an explanation why coaches emphasize the “follow-through” in many ball sports.

## Author summary

Accurate target-oriented throwing is a uniquely human behavior that, together with tool use, has been critical in human evolution. As temporal resolution in the neuromotor system appears crucial, previous studies identified a required accuracy of up to 1ms for the ball release. These reports conflict with estimates of neural spike train variability which is closer to 10ms. Novel analytical and behavioral results demonstrate that accurate performance is not limited by imprecise timing, but depends on hand trajectories that create temporal windows for ball release that are insensitive to variability and noise. These findings are invariant across tasks and highlight that humans overcome their temporal control limits by exploiting the geometry of the solution space, an apparent challenge to non-human primates and other animals.

## Introduction

As evolutionary biologists have noted, the primary advantage of human upright gait is that it frees the hands to hunt, fight, explore, and exploit the environment [1, 2]. Unlike other animals, humans have developed the ability to throw a projectile to hit a target, which significantly extended man’s area of influence [3–6]. The ability to throw, together with other forms of tool use, has co-evolved with, or even been instrumental to the development of cognitive abilities that have given humans their evolutionary advantage [6]. Importantly, while non-human primates can also throw objects, humans are vastly superior when accurately aiming at a target, as can be easily seen in ball sports. This disparity indicates an essential difference between coordinating one’s own movements and coordinating one’s body with respect to external reference points [7]. Given its demands on coordination, accurate throwing has become central in a host of sports disciplines, ranging from baseball to darts, and attracts children, athletes, and audiences alike. Hence, numerous studies in comparative and evolutionary biology, sports biomechanics, and motor neuroscience have tried to understand this quintessentially human skill [4, 8–11].

Additionally, accuracy and precision of timing in motor skills is interesting from a neuroscience perspective because it sheds light on the limitations of information transmission within the neuromotor system. How accurate or how variable are neural signals? What is their time resolution? The skilled handling of a projectile with the goal to hit a target requires an arm action culminating in the controlled release of the projectile at exactly the right moment. Ignoring the varying grasps needed for different “ball” shapes, the timing of release is more than just a response to an external cue: it critically depends on the ongoing arm movement. Even further, the distance and direction of the target significantly alters the demands on release timing and the thrower has to sensitively adjust his/her arm movements to the external reference to succeed. Several prior biomechanical analyses of human experiments reported that accurately throwing a ball at a target requires extremely precise timing of ball release, with an accuracy up to 1-2ms [9, 11–15]. These estimates are astonishing as timing variability in other tasks, such as rhythmic finger tapping, has rarely been reported below 10ms, even in professional musicians [16–18].

The ubiquitous variability is typically ascribed to intrinsic neuromotor noise arising from all levels of the sensorimotor system, ranging from ion channel dynamics to the timing of action potentials [19]. Further, the level of noise is widely assumed to increase with the magnitude of the signal, i.e., signal-dependent noise [20, 21] and the well-known speed-accuracy trade-off predicts higher variability in faster movements [22, 23]. Hence, fast and forceful movements as needed in throwing are expected to be highly variable. Nevertheless, reducing variability in timing is regarded as essential for improving accuracy and precision in throwing [8, 24]. How can a thrower be successful given the pervasive variability to meet the seemingly extreme timing requirements?

While learning a skill like throwing, it is widely acknowledged that although error and variability decrease with practice, variability is never completely eliminated. Previous work on a throwing task showed that humans reduce overt variability, including intrinsic neuromotor noise, but only to a limited degree [25–28]. However, the neuromotor system is highly redundant and multiple joint configurations can achieve the same goal [29]. Throwing presents a good example for this so-called motor equivalence as a target hit can be successfully achieved in infinitely many ways, due to the redundancy in the human body. However, the goal-directed action offers another level of redundancy arising from the task itself. Consider the bull’s eye in a dartboard. While the circular target area already allows for many dart trajectories to earn a full score, even more options arise when considering that each dart can hit the bull’s eye with different orientation angles. This task redundancy is the dual to the redundancy in the body, and opens up another infinity of equivalent solutions. In this study, we will show that some of these successful solutions are “smarter” than others because they require less precise timing. This may be related to what athletes and coaches in tennis and baseball refer to as “follow-through”: athletes should concentrate on the “follow-through” in their arm movements–even after ball impact as in tennis, or after ball release as in basketball. This instruction presents a puzzle as the arm trajectory after the ball release has no effect on the accuracy of the throw. Our results will provide a rationale for this coaching tradition.

This study examined the learning of a throwing skill in a simplified virtual throwing game that is similar to tetherball or the table game skittles. Mathematical analyses revealed that the task could be executed with infinitely many strategies that comprise a manifold of solutions. Additionally, the virtual set-up allowed for task variations where distance and direction relative to the thrower created significantly different geometries of the solution space. This posed qualitatively different requirements on the strategies that humans take to improve throwing performance. Virtual rendering permitted exact recording of the kinematic arm trajectory and, based on ball release timing, exact computation of the ball trajectory. Estimates of timing error were possible based on the mathematical analyses of the solution space. Critically, we were able to quantify the time for which the arm trajectory in each throw aligned with the solution manifold, rendering a timing window or a tolerance zone within which any ball release would achieve a successful target hit.

Four groups of human subjects practiced throwing to hit a target over six daily practice sessions. Each group had a different target location. Three target locations permitted strategies with extended timing windows, while one control location did not afford such a solution. These experiments tested four specific hypotheses:

***Hypothesis 1***: Performance, measured by the number of successful hits or the errors from the target, is expected to improve with practice. Importantly, however, both the amount and the rate of improvement depend on the geometry of the solution space as determined by the target location relative to the thrower.
***Hypothesis 2***: Timing error decreases with practice (Hypothesis 2A). This error has a lower limit that is most likely determined by the limited temporal resolution of the nervous system; therefore, it is independent of the task (Hypothesis 2B).
***Hypothesis 3***: Humans develop strategies with timing windows that afford tolerance to their intrinsic noise; this timing window increases with practice (Hypothesis 3A). Unlike timing error, this timing window has no specific upper limit, but both its limit and its rate of improvement is critically determined by the geometry of the solution space (Hypothesis 3B).
***Hypothesis 4***: Both timing error and window significantly contribute to performance. However, they develop at different rates.

## Results

**Fig 1:**
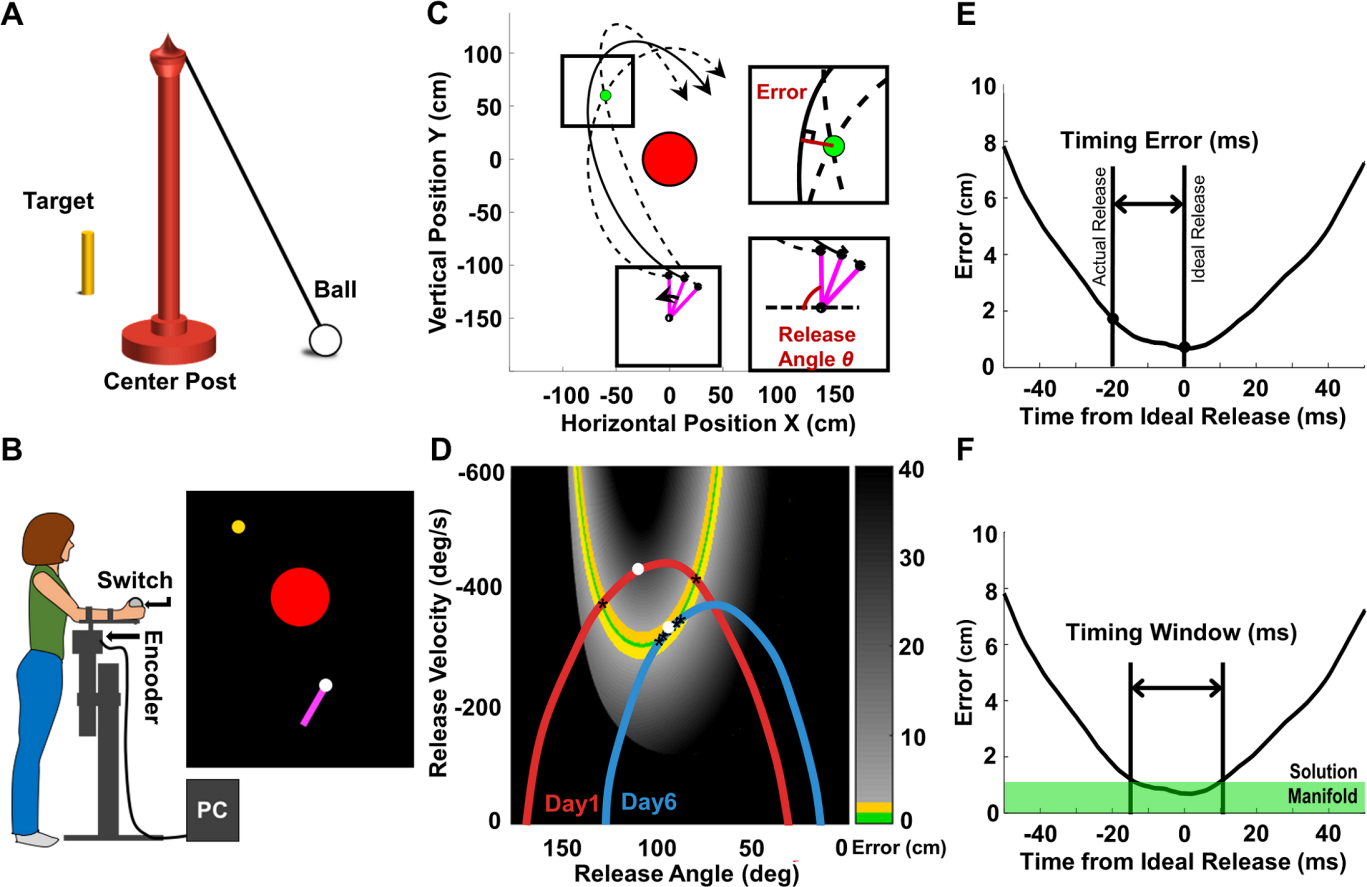
Model throwing task, experimental set-up and analyses of timing measures. **(A)** Real skittles task. **(B)** Experimental setup of the virtual throwing task; manipulandum and screen display. **(C)** Top down view of skittles workspace. Red circle is the post, green circle is target and pink bar represents the lever arm. The ball trajectory shown by the solid line incurred a non-zero error; the two dashed ball trajectories were successful throws hitting the target. The two insets illustrate the definition of the error around the target (top) and the release angle (below). **(D)** Execution space of the skittles task is spanned by two execution variables, release angle and velocity. Assuming each point in the space is a ball release, the error is calculated and shown in color code: green denotes the solution manifold, where each point leads to a hit below the error threshold of 1.1cm. The adjacent yellow band is the error threshold, denoting throws that hit the target but with an error larger than 1.1cm and smaller than 2.5cm (no intersection of ball trajectory with target). Two representative arm trajectories of the same subject are plotted in execution space, red from Day 1, blue from Day 6; white dots represent ball releases. The release on the red trajectory resulted in a large error; the release on the blue trajectory in a successful hit. **(E)** Each arm trajectory can be represented as an “error trajectory”, assuming each point on the trajectory is a ball release associated with an error. The timing error was calculated as the difference between the time of actual and ideal release. **(F)** Using the same “error trajectory” the timing window was quantified as the time that the trajectory would yield errors below the success threshold of 1.1cm, i.e. within the solution manifold.

Subjects threw a tethered ball around a post to hit a skittle at the far side of the post (Fig 1A). In the experiment, the task was rendered in a two-dimensional virtual environment, showing the workspace in a top-down view. Subjects manipulated a horizontal lever arm with a single-joint movement of the forearm to throw a virtual ball in the virtual workspace (Fig 1B and 1C). They were instructed to hit the virtual target (small yellow circle) without the ball touching the center post (larger red circle). Fig 1D illustrates the solution space of the task, which depicts the mapping between the two execution variables, angular position and velocity at release, onto the result variable, performance error. For each combination of release angle and velocity, an error was calculated from the motion equations for the ball (S1 Text). The error is marked by color, where errors for ball trajectories that did not hit the target, i.e. larger than the target radius (2.5cm, Fig 1C inset) are shown in grey; darker shades indicate higher error; black refers to errors larger than 40cm and post hits. Ball trajectories that struck the target, but had errors larger than the success threshold (1.1cm) are shown in yellow; the areas shown in green are successful target hits (errors<1.1cm). These successful hits were also signaled to subjects by a change of the target color from yellow to green, which served as reward feedback. This green curve is the set of solutions referred to as the “solution manifold”. The task has redundancy as there are infinitely many pairings of release angle and velocity that lead to successful target hits [10, 27, 30, 31].

**Fig 2:**
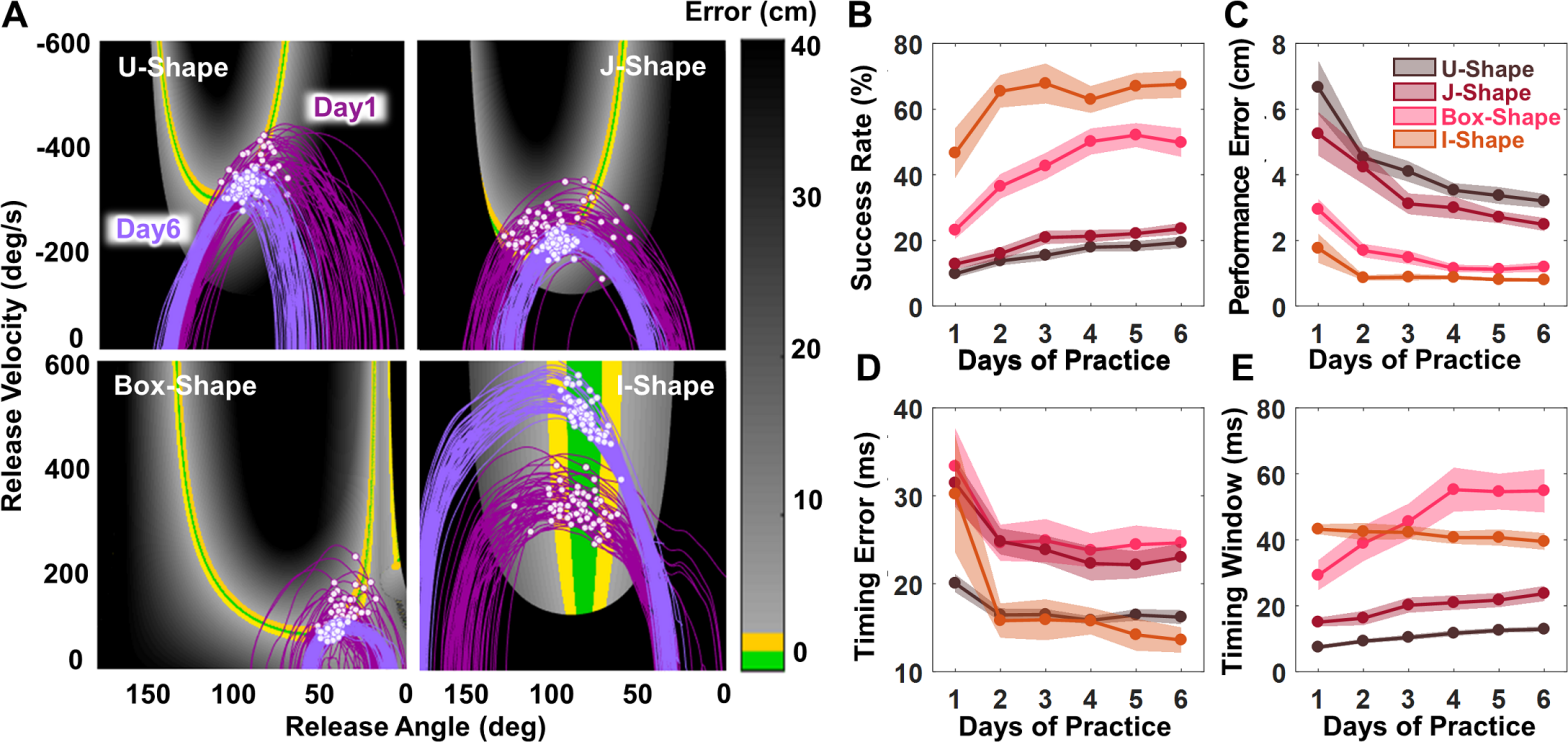
Solution spaces for the four different tasks and results of performance and timing measures for the four tasks. **(A)** Solution spaces with 60 exemplary arm trajectories and release points on Day 1 (dark purple) and Day 6 (light purple) in each of the four tasks. White dots represent the points of release. **(B)** Task success (percent of hits per day with 240 throws) across the 6 practice days of all subjects in the four tasks. **(C)** Average performance error across the six practice days of all subjects for each of the four tasks. **(D)** Average timing error across the six practice days of all subjects in four tasks. **(E)** Average timing window across the six practice days of all subjects in the four tasks. For all four figures the shaded regions represent ±1 standard error of the mean.

Four groups of 10 subjects practiced throwing to four different target locations, each for six daily sessions (see Methods, Table A in S1 Text). Based on mathematical analyses, these target locations were chosen to render different solution manifolds. Fig 2A shows these four solution spaces together with exemplary arm trajectories from one subject per task. The shape of the solution manifold differs significantly, and the tasks were referred to as U-Shape, J-Shape, Box-Shape, and I-Shape (Fig 2A). Note that not only the shape, but also the error gradient adjacent to the (green) solution manifold differed. This error surface determined the difficulty of the task and its success rate as formulated in *Hypothesis 1*.

Fig 2A shows 60 exemplary arm trajectories from one representative subject for each of the four solution spaces on Day 1 and Day 6. Notably, the points of release on Day 6 are more closely grouped around the solution manifold than on Day 1, reflecting that performance indeed improved with practice—as should be expected when practicing this novel task. A more subtle but critical change is that with practice, the arm trajectories “molded” to overlap more with the solution manifold, thus allowing for ball releases resulting in smaller errors. Four dependent measures quantified these changes: success rate, performance error, timing error, and timing window (see Methods).

### Task performance: Success rate and performance error

Two straightforward descriptive measures characterized overall task performance: success rate, defined as the percent of successful target hits, and performance error, defined as deviation of the ball trajectory from the target. As expected, Fig 2B and 2C show that success rate increased and performance error decreased over 6 practice days. Rendering each of the two variables to a separate two-way 6 (day) x 4 (task) analysis of variance yielded symmetrical effects. The main effect of practice day was significant for both success rate, F(3.09,111.31) = 40.10, p<0.001, and for performance error, F(1.60,57.42) = 52.34, p<0.001. The main effect of the four different tasks revealed significantly different magnitudes for success rate, F(3,36) = 57.03, p<0.001, and also for performance error, F(3,36) = 39.91, p<0.001. There were significant interactions for both success rate, F(9.28,111.31) = 4.293, p<0.001, and performance error, F(4.79,57.42) = 3.60, p = 0.007, indicating that each measure changed at different rates across the four tasks. Subsequent one-way ANOVAs confirmed that success rate and performance error significantly improved in all four tasks (see detailed results in Table A in S2 Text).

These findings strongly support *Hypothesis 1* and show how the magnitude and rate of performance improvements differed between tasks. As Fig 2B and 2C illustrate, the I-Shape task was the easiest with a hit rate over 60% after Day 2 and, consequently, the lowest error. Note that the I-Shape is a singular case as the solution manifold is parallel to velocity, such that release velocity does not matter for the success. In contrast, the U-Shape manifold presented the highest challenge and its success rate remained below 20%, even on Day 6; consequently, the error was highest. Such a stark contrast in task difficulty was surprising given the seemingly trivial differences in target locations (Table A in S1 Text, Fig 2A). These results clearly illustrated how a relatively small change in the direction and distance of the target relative to the thrower affected the geometry of the solution space and thereby critically determined the task difficulty.

### Timing measures: Timing error

The timing error of each throw measured the accuracy of release, and was defined as the difference in time between the actual and ideal release. Fig 1E shows the calculations on an exemplary “error trajectory”, which is generated by calculating the error associated with each angle and velocity pair along the arm trajectory, as if the ball had been released at each state. The minimum in the error trajectory denotes the ideal release time, where a ball release would result in the smallest error. The actual release typically occurred at a different time, leading to a non-zero timing error. For each practice day, the median timing error of the 240 throws characterized the timing error of each subject.

Fig 2D depicts the subject averages over practice days for each task, showing that timing error was higher for the Box-Shape and J-Shape tasks compared to the two other tasks. This relative ranking was not consistent with the two performance measures. Hence, timing error conveys independent information. Performing the same two-way ANOVA revealed not only the expected improvements in timing error with practice day, F(1.81, 65.00) = 18.45, p<0.001, but also significant differences between tasks, F(3,36) = 8.02, p<0.001; the interaction was not significant, F(5.42, 94.90) = 1.36, p = 0.25. These improvements supported *Hypothesis 2A* and were detailed in additional one-way ANOVAs for each single task (Table A in S2 Text). Further and more critically, Fig 2D suggests that timing error decreased quickly at the beginning and plateaued after Day 2 for all four tasks. Posthoc pairwise comparisons between practice days for each shape confirmed that timing error only significantly decreased from Day 1 to Day 2, without further change thereafter. Note that the I-Shape task showed the highest improvements in timing error and dropped to 13.60ms, compared to 23.00ms (J-Shape) and 24.65ms (Box-Shape) and 16.20ms (U-Shape task). Hence, the limits in timing error were *not* independent of the task, *counter to Hypothesis 2B*. Rather, the task set different limits to this quantity, at least for the six days tested in the experiment.

### Timing measures: Timing window

The timing window quantified the duration that the arm trajectory travelled parallel to the solution manifold within an error margin of 1.1cm (Fig 1F). As with the calculation of timing error, the arm trajectory was converted into an “error trajectory”, assuming each point in state space (angle and velocity) was a ball release. Plotting this error trajectory over time, the timing window quantified the duration on the trajectory during which a ball release would achieve successful hits (errors<1.1cm). Timing window was calculated for each trajectory and the mean value was computed for each practice day over the 240 throws of each subject.

Fig 2E depicts the average measures over practice days. The Box-Shape task showed the longest timing window, while the U-Shape task showed the shortest timing window. The rank ordering of values across tasks was different from the other dependent measures, again indicating independent information about the execution. The main effects of the 6x4 ANOVA documented that on average the timing window increased with practice, F(3.21,115.61) = 19.50 p<0.001. The significant main effect of task, F(3,36) = 34.94, p<0.001 and the significant interaction, F(9.63,115.61) = 9.47, p<0.001, revealed that the timing window was critically determined by the geometry of the solution space. On Day 6 the Box-Shape afforded the longest timing window of 54.84ms, improving from 29.22ms on Day 1. In contrast, in the U-Shape task subjects only improved from 7.43ms to 12.88ms across the six days (Table A in S2 Text). Notably, the timing window in the I-Shape did not lengthen with practice (p = 0.165), as the subsequent one-way ANOVA showed (see also Table A in S2 Text). With this one exception (to be discussed below), the findings supported *Hypothesis 3A*. The different magnitudes and rates of increase speak to *Hypothesis 3B*, which posited that the task geometry determines the degree to which timing window or tolerance to timing variability could be exploited.

### Relative contribution of timing error and timing window to task performance

**Fig 3:**
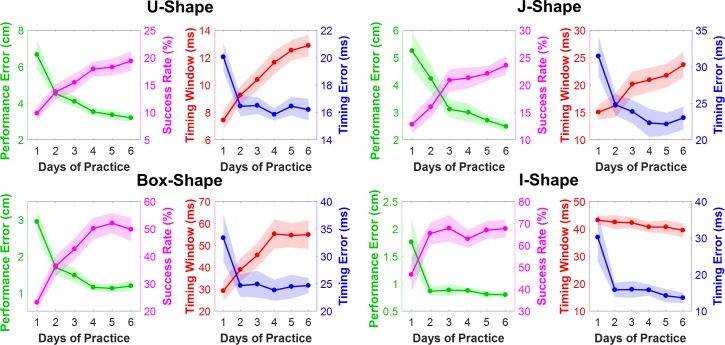
Results of performance and timing measures separated by task. Performance measures, success rate and performance error, are combined into one figure; timing error and timing window are combined into a separate figure. The units are scaled differently for each task to highlight the relative changes. Shaded regions represent ±1 standard error of the mean.

To reveal the pattern of changes in these measures, we also grouped them for each task and re-scaled them to their respective ranges (Fig 3). The two performance variables are presented together in one panel and the two timing variables are combined in a second panel for each task separately. As reported above, for all four tasks, success rate increased while performance error decreased. Less intuitive were the relative changes in timing error and timing window. For all four tasks, timing error dropped sharply on Day 1 and then leveled off, approaching a plateau by Day 6. In contrast, the timing window continuously increased in three of the four tasks. The one exception was the I-Shape task, where timing window did not change across the six days. The continuous increase in timing window closely correlated with the increase in the success rate. This pattern of change in the two timing measures suggests that at the beginning of practice, timing error improved until it reached a limit. Then, only an increase in the timing window contributed to further improvements in performance. These different rates of changes are consistent with *Hypothesis 4*.

**Fig 4:**
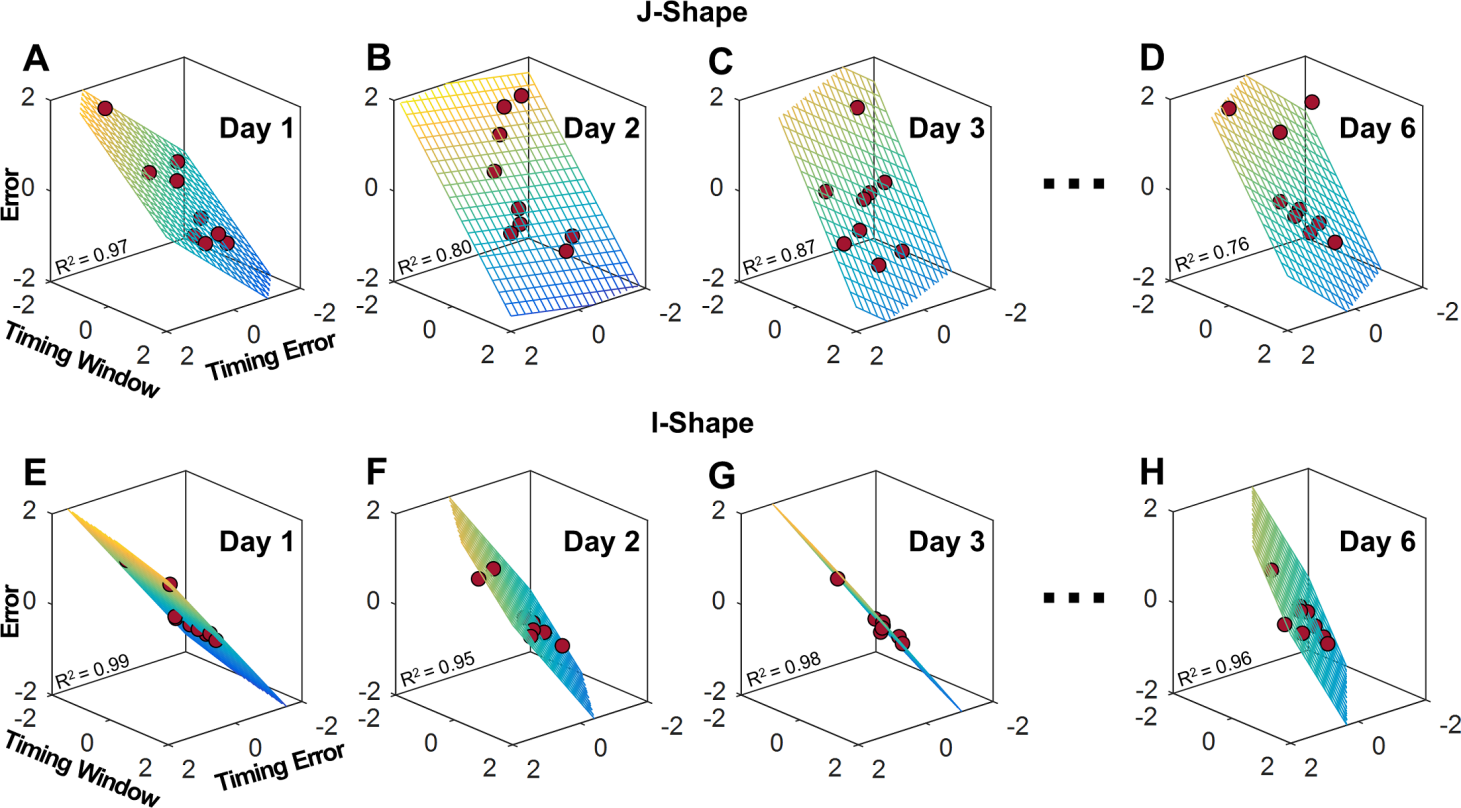
**Results from multiple regressions of performance error on timing error and timing window for the J-Shape (A-D) and the I-Shape (E-H) task across practice days.** Each dot represents performance of a single subject. The *x-*, *y-* and *z*-axis represent the normalized timing error, normalized timing window, and normalized error respectively. All variables were transformed into *z*-scores prior to each regression to make units comparable.

**Fig 5:**
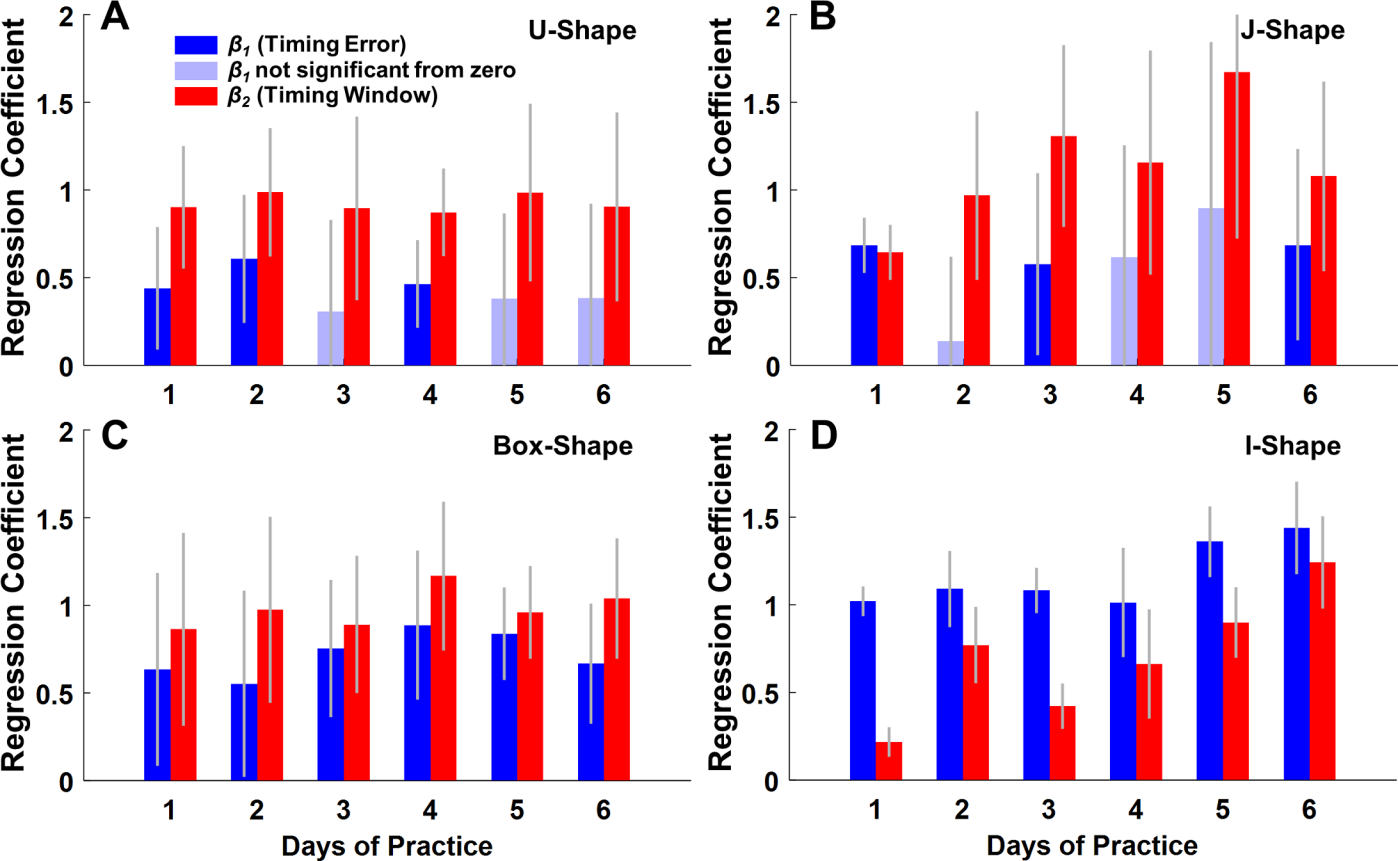
Results from multiple regressions of performance error on timing error and timing window. **(A)** Regression coefficients of the U-Shape task. Blue bars represent the values of the standardized regression coefficients of timing error (*β*_*1*_); the red bars represent the values of the standardized regression coefficients of timing window (*β*_*2*_). The gray error bars represent the 95% confidence intervals for *β*_*1*_ and *β*_*2*_. The light blue shaded bars indicate that the coefficients were not significantly different from 0 (p≥0.05). **(B)** Regression coefficients of the J-Shape task. **(C)** Regression coefficients of the Box-Shape task. **(D)** Regression coefficients of the I-Shape task.

To statistically confirm this invariant pattern, multiple linear regressions were conducted that regressed the performance error to the two timing measures. Fig 4 illustrates the regression planes for the J-Shape and the I-Shape task on Day 1, 2, 3, and 6; each point represents a single subject’s performance. The R^2^-value of all regressions ranged between 0.69 and 0.99, with most values close to 0.85. Comparing the regressions planes across days reveals that for the J-Shape the planar angle displayed a noticeable rotation from Day 1 to Day 2, but remained similar from Day 2 to Day 6. This qualitative change was quantified by the (standardized) regression coefficients, *β*_*1*_ for timing error and *β*_*2*_ for timing window, summarized in the bar charts of Fig 5. For the J-Shape task, *β*_*1*_ and *β*_*2*_ had similar values on Day 1, but on subsequent days the *β*_*2*_-values were always higher than *β*_*1*_-values. On Day 2, 4 and 5, *β*_*1*_ was no longer significantly different from zero. Those results suggest that after the first practice day, the performance error became more reliant on timing window than timing error, indicating a significantly different contribution to performance, consistent with *Hypothesis 4*.

Similar patterns could be found in the regression results of the U-Shape task (Fig 5A). After the second practice day, the significance of the contribution of timing error dropped, while timing window remained a significant predictor of performance error across all days. In the Box-Shape task (Fig 5C), timing error and timing window both significantly predicted the error across 6 days, however *β*_2_ was consistently larger than *β*_1_ on all 6 days. Additional analysis did not detect any significant collinearity between timing window and timing error, confirming that these two measures provide independent information.

However, not all tasks afforded this tolerance to variability. The I-Shape task was selected as a control condition, where the solution manifold ran parallel to velocity, i.e. the results were insensitive to velocity (Fig 2A). While all other tasks allowed subjects to align their arm trajectory with the solution manifold, performance of the I-Shape task was solely determined by release angle. Therefore, timing error was more important than timing window as visible from the regressions (Fig 4E–4H). In this task, the regression coefficient *β*_*1*_ was consistently larger than *β*_*2*_, reflecting that timing error was more important than timing window (Fig 5D). This result is the consequence of the fact that it was physically impossible for subjects to align their arm trajectory with this manifold–it would require infinite acceleration.

To conclude, the regression analyses revealed that timing error and timing window contributed to a different extent to performance, supporting *Hypothesis 4*. More specifically, timing error was improved first in practice and timing window continued to be improved during extended practice, with the exception of the I-Shape task. These findings strongly suggest that creating a window for timing is the critical determinant for skilled performance, as it affords tolerance to imprecision due to noise in timing.

## Discussion

The ability to throw a projectile to a target has been an essential step in the evolution of mankind [1, 2]. Today, accurate throwing has become quintessential to numerous ball games and athletic disciplines and has held the fascination of athletes and audiences for centuries. Non-human primates and other animals can manipulate objects, but they are unable to aim at a target in the environment, except under unusual circumstances [4, 32]. While throwing by itself requires the coordination of the multi-segmented body in an egocentric reference frame, aiming to a target requires orienting one’s own bodily actions to an allocentric reference frame [33, 34]. As our findings demonstrated, it is the fine-tuning of the arm trajectory and creating a longer window for successful ball releases that critically determine a dexterous throw. Hence, timing of the ball release is important, but throwing is not “*all* about timing”.

Guided by four hypotheses, our results showed that the error and successful hits critically depended on the geometry of the solution space defined by the target location relative to the thrower (*Hypothesis 1*). Timing error decreased with practice, but plateaued relatively early (*Hypothesis 2A*). While it is reasonable to expect that this may reflect a limit of the nervous system, this plateau was different for the four tasks, at least after six days of practice (*counter Hypothesis 2B*). This result suggests that this timing inaccuracy may not be caused by a limit of the nervous system, but rather determined by the solution geometry that affords an alternative strategy of creating a tolerance zone. Consistent with this later conjecture, the timing window steadily increased with practice, without an apparent limit (*Hypothesis 3A*). Instead, this timing tolerance was also critically determined by the geometry of the solution space (*Hypothesis 3B*). Finally, timing error improved significantly in early practice, while during extended practice the timing window was the major determinant of the outcome (*Hypothesis 4*). While absolute values of error and timing measures differed, this pattern was invariant across tasks, except the I-Shape that presents a singular control case. These results suggest that while the unavoidable neuromotor noise sets limits to precision and accuracy of timing, humans exploit the task redundancy to develop strategies that make their noise matter less [35].

### Geometry of the solution space determines task difficulty

What creates the challenge when throwing to hit a target? To begin, task difficulty does not only depend on the distance between target and thrower, as long as strength and contraction speed of the muscles do not reach physiological limits. Even though Venkadesan and Mahadevan [10] proved that noise is amplified in longer flight durations, our results showed that the target with the longest distance from the thrower (I-Shape task) had the smallest performance error, while the task with the shortest target distance (U-Shape task) had the largest error. These results highlight that it is the *relative* location of the target to the thrower that determines the solution space and its noise-sensitivity. Evidently, subjects were unaware of the mapping from workspace to solution space and could only probe this mapping by trial and error. Further, each throw is likely to be executed open-loop as the movements are very fast. Note also that exploration across trials via following an error gradient in solution space can only be successful in neighborhoods close to the solution manifold. The solution spaces in the present conditions show a set of minima and discontinuities that would pose significant problems to simple gradient descent algorithms [36, 37].

Our data clearly showed that the performance and its rate of improvement were highly dependent on this geometry (*Hypothesis 1*). Interestingly, the timing errors, measured in milliseconds, were lowest for both U- and I-Shape tasks, even though task success was highest in the I-Shape task and lowest in the U-Shape task. The timing window showed yet a different rank ordering. The independent contributions of these different variables strongly suggest independent means to achieve the task. Their relative contributions can only be understood on the basis of the solution space. Note that, with the exception of the study by Venkadesan and Mahadevan [10], none of the previous studies examined throwing with different target orientations.

Analysis of the timing window revealed that task difficulty is determined by how the arm trajectory can be oriented with respect to the solution manifold. Creating long timing windows requires a geometric match between the inverted U-shaped arm trajectory in state space and the solution manifold. This matching is partly constrained by biomechanical factors, i.e. can the invariably U-shaped trajectory be shifted and shaped to align with the manifold? An extreme case is the I-Shape task, which is the singular case where the U-shaped manifold collapses into the one line. Here, the arm trajectory needs to have infinite acceleration; this is of course physically and biomechanically impossible. Therefore, the timing window should not and did not increase in the I-Shape task. To improve performance for the I-Shape manifold, the only avenue is to improve the timing error. Conversely, while the U-Shape task was difficult and errors remained high, the timing window lengthened continuously throughout the six days and there were no signs of a plateau, showing potential for further improvements with practice.

### Limits in timing precision and neural information transmission

Consistent with *Hypothesis 2*, there was an asymptote in timing error, although this limit differed between tasks, ranging between 14 and 25ms. These differences between tasks may be due to the limited practice time or due to the different geometries afforded different timing window to be exploited. The values are consistent with a previous report in a similar throwing task where experts could not improve their timing error beyond 9ms, even after 15 practice days [38]. However, these estimates of timing accuracy differ from several other studies on throwing, which reported much shorter times. For example, both Nasu et al. [11] and Smeets et al. [13] recorded 3D kinematics of dart players and estimated timing accuracy as low as 1-7ms. However, due to the limitations of recording in a realistic setting, Smeets et al. [13] only recorded the subject’s thumb trajectory and final errors directly, while the trajectory of the dart was inferred. Nasu et al. [11] determined the timing window assuming that the finger’s velocity is identical to the dart’s velocity. However, in this unconstrained setting, the velocity of the ball at release is necessarily higher than the finger velocity to separate the projectile from the finger. As the timing analyses are conducted on the finger trajectory, this leads to systematic errors in the dart trajectory. This is evident in the relatively large differences between the computed and measured errors at the bull’s eye, which is in the order of the performance error itself. Therefore, the reported timing precision is likely to be an under-estimation.

A muscle-biomechanical study by Chowdhary and Challis [9] suggested that humans needed 1-4ms timing precision for a successful throw. However, their estimates rested on several strong assumptions and a control policy that used flight duration as a cost criterion. However, Venkasedan and Mahadevan [10] demonstrated that ball trajectories with higher speed are more sensitive to noise, which can compromise accuracy. Note that flight duration and speed are not a criterion in accurate throwing per se. Speed is only required when there is an opponent or a goalkeeper who tries to intercept the ball or when the projectile has to hit the target with high speed, such as a spear for hunting prey [12]. Finally, several studies by Hore and colleagues estimated timing precision with respect to selected kinematic landmarks, such as the hand at its vertical position [8, 39]. While reasonable, the reference to a landmark assumes that the hand trajectory is invariant, an assumption that is unrealistic as seen in our and other results. Given the minute temporal estimates, such variations may critically affect the calculations.

In contrast to these studies, our analyses utilized a reference point for each throw based on the analytic understanding of the task and its solution manifold. As the optimal release time could be calculated accurately for each individual trajectory, our calculations did not have to rely on threshold values. Further, in the virtual release the release velocity of the ball did not differ from the hand velocity, as the fingers did not impart momentum to the projectile to separate it from the hand. Constraining the arm trajectory to a single-joint rotation eliminated the need to model or approximate the arm trajectory; lastly, the virtual environment had no external perturbations, such as air resistance, ball spin, and complex measurement issues due to finger movements at ball release [13, 15]. We submit that these methodological advantages render our estimates of timing precision reliable, at least for these controlled throwing conditions.

Our estimates of timing error are on the same order of magnitude as those reported in the literature on rhythmic timing. When tapping to a metronome, the standard deviations of the period are usually around 5% of the tapping period. Testing professional musicians on periods of 500ms, Repp [18] reported standard deviations of 11ms, while non-musicians tapped with standard deviations of 15ms. In a perception study, Repp and Steinman [40] found that for melodies with a 200ms baseline period, musicians could detect changes with 65% accuracy when intervals were lengthened by 8ms or shortened by 9ms. When timing was tested with respect to an external cue, again a limit of no less than 7ms has been reported: extensive practice (80,000 repetitions!) to tap 460ms after a visual cue left the tapping experts with a standard deviation of 7ms [41].

These estimates for limits in timing accuracy are also consistent with experimental and theoretical studies of variability in the firing rates of neurons. Cortical neurons, measured in a rhesus monkey, have firing rates between 15–220 spikes/s. Even during portions of consistent neural recordings, the inter-spike intervals varied significantly: a mean inter-spike interval of 7.35ms had standard deviations of 5.28ms [42]. To obtain accurate signal transmission, one could theoretically “average away” the variability in a single neuron’s spike rate over a large population. However, simulation studies showed that this averaging is limited because neighboring neurons tend to synchronize and the variability between neurons is correlated [43]. Therefore, averaging even large numbers of neurons will only yield an estimate that is 2–3 times better than the accuracy of measuring a single neuron [44, 45]. In an ensemble of weakly correlated neurons, Mazurek and Shadlen [43] showed that a signal that changes faster than 10ms (100Hz) could not be distinguished from noise. While these estimates may differ for sensory events, for example, a primate study on the visual system reported a temporal precision of neural coding of 2-10ms [46], a higher resolution in sensory signals does not overcome the resolution limits in motor signals. Taken together, these findings suggest that timing in the motor system is limited to an accuracy in the order of 10ms.

### Timing window, state-dependent timing and task affordance

While timing error quantifies the bias for each throw, timing window is a model-based measure that reveals how much variability can be tolerated without affecting the performance. As expected, the actual durations varied with the task, as this measure results from the matching of the arm trajectory to the geometry of the solution manifold. For example, the U-Shaped manifold only allows a window of 15ms, while the Box-Shape manifold allows 50ms, depending on how subjects can shape their arm trajectory. The extreme example is the I-Shape manifold, where biomechanics makes this matching impossible. These considerations present a new viewpoint on throwing, consistent with Gibson’s notions of affordance [47]: it is the geometric match between the task’s affordance with the thrower’s biomechanics that defines the difficulty of the task.

This analysis also stresses the essential difference between throwing and many other timing studies: in throwing, the timing of ball release depends on the states of the system. Therefore, it is arguably more complex than timing the onset of a movement to a visual or auditory cue [46, 48]. Rather, timing of release is integral to the dynamics of the movement and the task. However, could the task analysis and our results be an artifact of the choice of the coordinate system?

### Timing sensitivity is independent of coordinates

**Fig 6:**
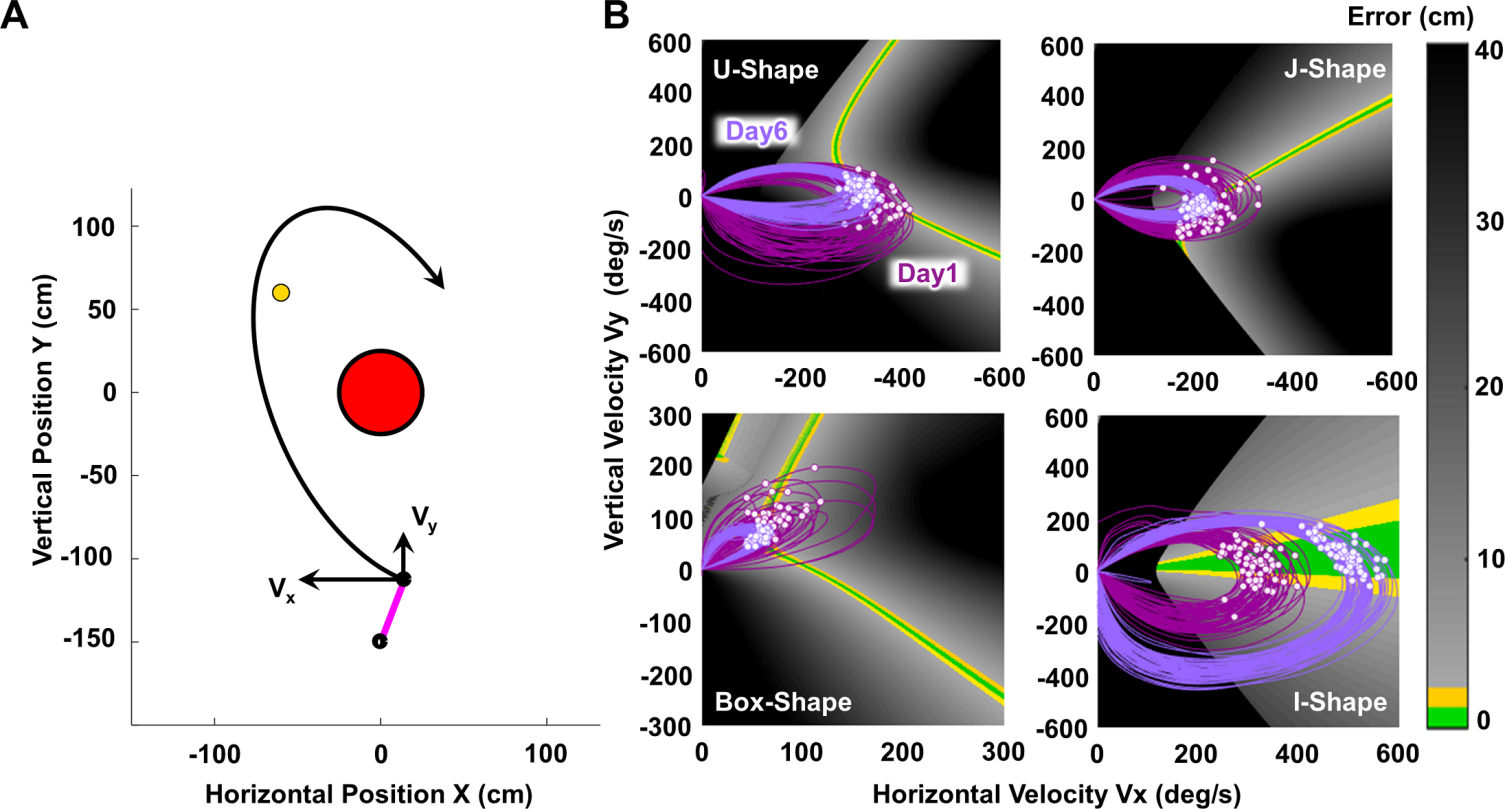
Skittles tasks represented in Cartesian coordinates. **(A)** Work space of the skittles task with the two execution variables defined in Cartesian coordinates. The variables are now velocity in orthogonal *x*- and *y*-directions and also fully define the trajectory of the ball. **(B)** Solution spaces of the four tasks spanned by the two Cartesian velocity variables together with the same exemplary arm trajectories as in Fig 2. The nonlinear transformation of the variables also transforms the solution manifolds and the arm trajectories. However, as can be seen from the release points, their errors remain unaffected.

Given the physical model of tetherball, the coordinates chosen for our analysis were not the only ones possible. In fact, changing the coordinate system can significantly influence the geometry of the solution space and any data distribution plotted in this space [49]. For the same physical model, the arm trajectory and the ball release can also be written in Cartesian *x-y* coordinates of the workspace, as shown in Fig 6A (see also Fig A in S3 Text). Tangential velocities at ball release expressed as components in the *x-y*-direction are alternative execution variables that fully determine the ball trajectory and its error from the target. Interestingly, the solution spaces spanned by these two execution variables showed very different shapes for the four tasks (Fig 6B): not only is the solution space rotated clock-wise by approximately 90 degrees, but the curvature of the error surface is also distorted. However, the arm trajectories underwent the same nonlinear transformations such that the ball releases on these arm trajectories retained their temporal distance from the solution manifold. Calculations proved that timing error and window were invariant under this transformation. This fact is worth noting as the choice of coordinates has been shown to critically determine the shape of the data distributions and therefore the results of covariance-based analyses [49]. The two timing measures in this study are free from such dependency.

### The importance of “follow-through”

How do subjects navigate this space of solutions and learn to shape their arm trajectory appropriately? A final comment should go to coaches and their emphasis on the “follow-through” in various ball sports, ranging from baseball to tennis: athletes should pay attention to their arm trajectory *after* they released or hit the ball [50, 51]. At first sight, this appears puzzling, as the arm trajectory after the ball release (or hit in a tennis serve) has no effect on the ball trajectory and its desired accuracy. Our results provide a justification for this seemingly meaningless exhortation: as the timing window determines the tolerance for variability, and consequently the accuracy of the ball, creating such a window requires shaping the arm trajectory *before and after* the critical release moment. Due to arm dynamics, generating a trajectory leading up to a successful ball release also requires shaping the trajectory *after* the single moment, as a trajectory cannot change abruptly. Hence, the athlete does indeed need to focus on the entire trajectory, including the segment that follows the ball release. Coaches may indeed have had the right intuition all along.

## Methods

### Ethics statement

All subjects gave informed written consent before the experiment and received monetary compensation upon completion of all practice sessions. The protocol complied with the Institutional Review Board of Northeastern University.

### Participants

Forty right-handed healthy individuals (21 males, 19 females, average age 21±2.4 years) participated in this experiment. None of the subjects had any prior experience with the experimental task.

### Task and apparatus

**Fig 7:**
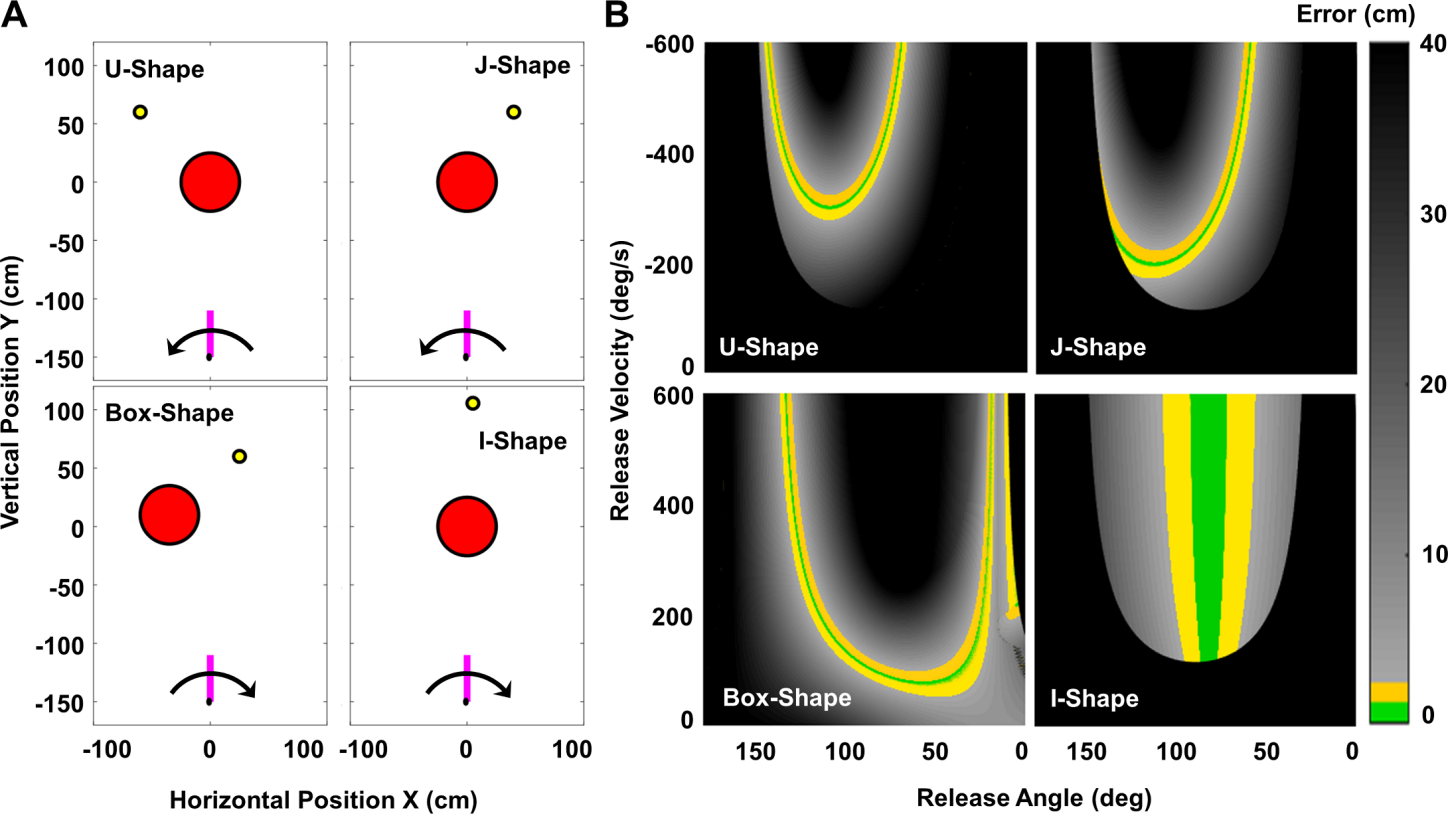
Workspaces and solution spaces of the four tasks. **(A)** Workspaces of the four tasks. The red circle in the middle is the post, the yellow circle is the target, and the pink bar is the lever arm; the black arrow indicates the direction of the arm movement. Each task has a different target location. The Box-shape task also located the post away from the center (0, 0 cm). **(B)** Solution spaces of the four tasks. Each of the four panels corresponds to the four workspaces in panel A. The error of all combinations of release angles and release velocities were calculated and coded by color; green denotes the set of points that hit the target with errors smaller than 1.1cm; yellow denotes errors larger than 1.1cm and smaller than 2.5cm; grey shades represent errors larger than 2.5cm; black denotes throws when the error is larger than 40cm or the ball trajectory hit the post.

The experimental task was modeled after the British pub game, skittles, or the American game, tetherball. The player throws a ball tethered to a center post to hit a target skittle on the other side of the post (Fig 1A). In the experimental task, the subject manipulated a horizontal lever arm with its axle at the elbow to throw a virtual ball in a 2D virtual environment (Fig 1B). The virtual environment displayed a top-down view of the workspace and was projected onto a large screen 150cm in front of the subject (Fig 1C). On the screen, the subject saw the post (red circle, radius = 25cm) in the center of the workspace (origin; 0,0 cm), a virtual lever arm (purple bar, length = 40cm) with its pivot located 150cm below the origin, the target (yellow circle, radius = 2.5cm) placed on the far side of the post, and the ball (white circle, radius = 2.5cm). The location of the post and the target varied across the four tasks, as shown in Fig 7A and detailed in Table A in S1 Text. Specifically, four different target locations were chosen to present different challenges (Fig 7B).

Subjects were instructed to throw the ball either from the lateral to medial side in clockwise direction (U-Shape and J-Shape tasks) or from medial to lateral side in anti-clockwise direction (Box-Shape and I-Shape tasks) to hit the target center without touching the post. To start each throw, the subject grasped a wooden ball affixed to the distal end of the lever and gently pressed his/her index finger on a force sensor (Interlink Electronics, Camarillo, CA). This attached the virtual ball to the virtual lever arm. The subject then moved his/her arm and released the ball by lifting the index finger off the force sensor. Upon release, the ball traversed an elliptical path in the virtual scene; its path was fully determined by angle and velocity of the lever arm at the moment of release (S1 Text). The arm angle was measured with a digital encoder (BEI Sensors, Goleta, CA) at 1kHz (USB-6229-BNC, National Instruments, Austin, TX). Angular velocity was estimated by the slope of a linear fit on the 20 angle samples over time prior to release. Error for a given throw was defined as the minimum distance between the ball path and the target center (Fig 1C). When the error was below 1.1cm, the target changed color from yellow to green to signal a successful target hit.

A two-dimensional model in which the ball was attached to two orthogonal massless springs with their rest position at the origin generated the ball trajectory for each throw. The equations for the ball position in the *x*-*y*-directions at time *t* were:

$$x(t)=A_{x}sin(\omega t+\varphi_{x})e^{-\frac{t}{\tau }}$$ (1)

$$y(t)=A_{y}sin(\omega t+\varphi_{y})e^{-\frac{t}{\tau }}$$ (2)

The frequency ω denotes the natural frequency of the springs, and the exponential term with time constant τ added damping to approximate realistic ball flight (details in S1 Text). ω and τ were different in the four tasks (Table A in S1 Text). The amplitudes *A*_*x*_, *A*_*y*_, and phases φ_*x*_ and φ_*y*_ were calculated from the measured position and velocity at ball release. Due to the restoring forces proportional to the distance of the ball from the origin, the ball traversed an elliptic path and was accelerated toward the origin.

### Redundancy and geometry of solution space

The essential feature of this task is that it has redundancy: while the position and velocity at release fully determine the ball trajectory and thereby its error at the target, there are a mathematically infinite number of solutions that achieve the same error. This set of solutions is referred to as the solution manifold. Fig 7A and 7B visualize this feature: for each target location, there exists a solution space spanned by release angle and release velocity. For each ball release, defined by its release angle and velocity, an error was calculated that is represented by color. Green denotes successful hits with errors <1.1cm, the solution manifold, yellow denotes errors <2.5cm, black denotes those ball releases that led to errors larger than 40cm or that hit the center post. The solution space can also be seen as a three-dimensional surface with the solution manifold at its minimum; the different curvature around the solution manifold represents its sensitivity or tolerance to intrinsic noise and control errors [28].

For the four target locations, successful hits form a solution manifold with different topological features: the U-Shape manifold has wider error tolerance for lower velocities; the J-Shape solution manifold transitions discontinuously into post hits, note that the curvature around the solution manifold decreases towards this discontinuity, indicating increasing tolerance to error while also increasing the risk for a post hit; the Box-Shape solution space has large areas of light grey indicating a shallow gradient around the solution manifold; additionally, the solution manifold reaches into much lower velocities; the I-Shape solution manifold runs parallel to velocity axis, indicating that throws and the resulting errors are insensitive to the velocity at release; additionally, it has a relatively wide solution manifold indicating that successful hits can be achieved with a range of release angles.

### Experimental conditions and dependent measures

Forty subjects were randomly assigned into one of four groups. Each group practiced the virtual throwing task with one target location for 6 days, with 240 throws per day separated into 4 blocks of 60 throws each. Thus, there was a total 1440 throws for each subject collected over 1–2 weeks. The four target constellations are shown in Fig 7A with their respective solution spaces in Fig 7B. The specifications of the target locations are listed in Table A in S1 Text.

#### Measures of task performance: Success rate and performance error

A throw was successful if its error was below the threshold, and the color change signaled a symbolic reward to the subject. Hence, a first measure was success rate, which was defined as the percentage of successful throws per daily session (240 trials). A more fine-grained variable was the performance error, measured as the minimum distance between the ball trajectory and the target (Fig 1C). As error was always positive and did not show a normal distribution, task performance on each day was characterized by the median error.

#### Measures of execution: Timing error and timing window

For each throw, the timing error and the timing window were computed. As shown in Fig 1D, the solution space spanned by angle and velocity not only presents the error or result surface, but was also the state space for the arm trajectory. Hence, the arm trajectory could be plotted in the same space and related to the solution manifold. Fig 1D shows two exemplary arm trajectories plotted in the solution, or state space. The red trajectory crosses the solution manifold twice, while the blue trajectory aligns more with the manifold; the white points mark the ball releases and the black asterisks represent all releases that can hit the target; the red trajectory incurred a high error, while the blue trajectory achieved a target hit. For successful hits, the release needs to be timed ideally at the intersection or as close as possible to the solution manifold. The difference between the actual release time and the time of the intersection quantified the timing error for a given trajectory. Further, the longer the trajectory aligned with the solution manifold, the longer was the timing window, i.e. the interval when a ball release would result in successful target hits.

To calculate these measures, each arm trajectory was first converted from a sequence of angle-velocity pairs to a sequence of errors, assuming each time point of the arm trajectory as a ball release (Fig 1E). For this error trajectory, the ideal release time was the moment with the lowest error, set to zero for reference. The timing error was defined as difference in time between the actual release to the ideal release for a given arm trajectory, measured in milliseconds (Fig 1E). In cases where there were two intersections, as exemplified by the red trajectory, the time between the actual release time to the closest crossing point was used. Due to the non-normal distribution, timing error for each practice day was quantified by the median of all throws.

The timing window for each throw was defined as the time where the error magnitude was less than 1.1cm, i.e. within the solution manifold (Fig 1F). If the arm trajectory crossed the solution manifold twice, only the segment close to the ideal release time was considered. An increase in timing window indicates that the arm trajectory became more tolerant to noise and errors. The timing window on each day of practice was quantified by the mean of all throws.

We also performed alternative calculations of timing error where the optimal release was set to be in the middle of the timing window. Even though timing error was no longer independent of the timing window, this alternative calculation did not produce any differences in the results.

### Statistical analyses

To characterize how the dependent measures changed across practice in the 4 different tasks, a 6 (day) x 4 (task) repeated-measures ANOVA with experimental task as a between-subjects factor and practice day as a within-subjects factor was conducted on each measure. The Greenhouse-Geisser correction was applied to the within-subject effects where sphericity was violated [52]. For all dependent measures, subsequent one-way repeated-measures ANOVAs examined the changes across the 6 practice days for each task. For the two timing measures, posthoc pairwise comparisons were applied between each practice day.

Multiple linear regressions assessed how timing window and timing error predicted performance error for each task separately: *y* = *β*_1_*x*_1_ − *β*_2_*x*_2_, where *y* was performance error, *x*_1_ was timing error and *x*_2_ was timing window. *β*_*1*_ and *β*_*2*_ were standardized regression coefficients of timing error and timing window. All variables were converted to *z*-scores prior to the regressions to accommodate for differences in the units.

## Supporting information

S1 Text Physical model of the virtual skittles task. Physical model, equations of motion and parameters of the experimental tasks.
(DOCX)

S2 Text Statistical results on task performance measures. Additional one-way ANOVA results of the four dependent measures.
(DOCX)

S3 Text Invariance to coordination transformations. Transformation from polar coodinatates to Cartesian coordinates of the workspace.
(DOCX)
