# Supplementary material for: Exploiting the geometry of the solution space to reduce sensitivity to neuromotor noise
Source: PLoS Comput Biol. 2018 Feb 20;14(2):e1006013. doi: 10.1371/journal.pcbi.1006013 (PMC5834204; doi:10.1371/journal.pcbi.1006013)
Supplement: S1 Text — Physical model, equations of motion and parameters of the experimental tasks. (DOCX) [file pcbi.1006013.s001.docx]

**Supplement 1:** **Physical Model of the Virtual Skittles Task**

The three-dimensional real skittles or tetherball task was simplified to a two-dimensional model by taking a top-down view on the task. Hence, the ball was modeled as suspended by two orthogonal, massless springs with its equilibrium state at the post location (Fig A).


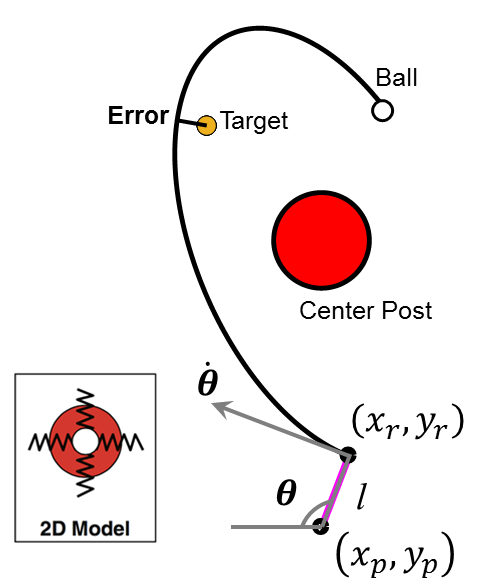


**Fig A.** Two-dimensional model of the skittles task as implemented in the virtual set-up.

With the measured angular position *θ* and velocity $\dot{\theta}$ at the time of release of the force sensor, the position and velocity of the ball at release in *x*- and *y*-coordinates were calculated as follows:

$x_{r}=x_{p}-l\cos\theta$ (S1.1)

$y_{r}=y_{p}+l\sin\theta$ (S1.2)

$\dot{x_{r}}=\dot{\theta}l\sin\theta$ (S1.3)

$\dot{y_{r}}=\dot{\theta}l\cos\theta$ (S1.4)

where *l* denotes the length of the lever arm, (*x*_p_ ,*y*_p_) denotes the pivot of the lever arm, and (*x*_r_ ,*y*_r_) denotes the states of the ball at release. With a given ball mass *m*, spring constant *k*, and relaxation time *τ*, the natural frequency *ω* of the system was calculated as:

$\text{ω}\text{ }\text{=}\sqrt{\frac{k}{m}-\left( \frac{1}{\tau} \right)^{2}}$ (S1.5)

where $\tau=\frac{2m}{c}$ , and *c* is the damping ratio. In this study, *c* was set to 0.01 for all tasks. The values for *m* and *k* differed between the tasks (see Table A).

After release, the ball trajectory followed the equations of motion:

$x\left( t \right)=A_{x}\sin\left( \omega t+\varphi_{x} \right)e^{-\left( \frac{t}{\tau} \right)}$ (S1.6)

$y\left( t \right)=A_{y}\sin\left( \omega t+\varphi_{y} \right)e^{-\left( \frac{t}{\tau} \right)}$ (S1.7)

where $\varphi_{x}$ and $\varphi_{y}$ were the phases at the time of release, and *A_x_* and *A_y_* were the amplitudes of the sine function in the *x*- and *y*-directions of the work space:

$A_{x}=\sqrt{x_{r}^{2}+\left[ \frac{\dot{x}_{r}}{\omega}+\frac{\left( \frac{x_{r}}{\tau} \right)}{\omega} \right]^{2}}$ (S1.8)

$A_{y}=\sqrt{y_{r}^{2}+\left[ \frac{\dot{y}_{r}}{\omega}+\frac{\left( \frac{y_{r}}{\tau} \right)}{\omega} \right]^{2}}$ (S1.9)

$\varphi_{x}=arccos\left[ \frac{1}{A_{x}}\left( \frac{\dot{x}_{r}}{\omega}+\frac{\left( \frac{x_{r}}{\tau} \right)}{\omega} \right) \right]$ (S1.10)
$\varphi_{y}=arccos\left[ \frac{1}{A_{y}}\left( \frac{\dot{y}_{r}}{\omega}+\frac{\left( \frac{y_{r}}{\tau} \right)}{\omega} \right) \right]$ (S1.11)

If the ball trajectory hits the post, the performance error was marked as 1. Such errors occurred in less than 1% of the trials. As we calculated the median for the performance error, these high values had negligible effect on the summary measures. Note that the post location for the calculation of the ball trajectory was at the origin for three tasks. Only in the Box-Shape task, the visible post location was shifted and presented as an obstacle to the left side (see Table A and Fig 7 in the main text). Note that the Box-Shape required to also change the ball mass and spring constant. The equations of motion for the ball trajectory were calculated in the same way for all four tasks. The four task conditions with different geometries of the solution space were generated by locating the target in four different *x-y* positions.

**Table A.** Parameters of the experimental tasks.

|  | Target Location  x, y (cm) | Physical  Post Location  x, y (cm) | Visual Post Location  x, y (cm) | Ball Mass m (kg) | Spring Constant k (kg/m) |
| --- | --- | --- | --- | --- | --- |
| **U-Shape** | -60, 60 | 0, 0 | 0, 0 | 0.1 | 1 |
| **J-Shape** | 40, 60 | 0, 0 | 0, 0 | 0.1 | 1 |
| **Box-Shape** | 25, 60 | 0, 0 | -35,10 | 0.5 | 3 |
| **I-Shape** | 5, 105 | 0, 0 | 0, 0 | 0.1 | 1 |
