# Supplementary material for: Exploiting the geometry of the solution space to reduce sensitivity to neuromotor noise
Source: PLoS Comput Biol. 2018 Feb 20;14(2):e1006013. doi: 10.1371/journal.pcbi.1006013 (PMC5834204; doi:10.1371/journal.pcbi.1006013)
Supplement: S2 Text — Additional one-way ANOVA results of the four dependent measures. (DOCX) [file pcbi.1006013.s002.docx]

**Supplement 2: Statistical Results on Task Performance Measures**

For the two performance measures, success rate and performance error and the two execution measures, timing error and timing window, additional one-way ANOVAs were conducted to scrutinize the changes across practice days. Means and standard deviations of the error and timing measures on Day 1 and Day 6 are tabulated below, together with the statistical results of the one-way ANOVAs. As Table A overviews, success rate and performance error significantly improved in all four tasks, as to be expected (*Hypothesis 1*). Timing error decreased in all four tasks, although the drop in the I-Shape task was highest from 30ms to 13ms, consistent with *Hypothesis 2*. The timing window lengthened in three tasks, consistent with *Hypothesis 3*. As expected, there was no increase in the I-Shape task due to the vertical orientation of the solution manifold.

**Table A.** Statistical results of the four dependent measures.

|  | Day 1 (M, SD) | Day 6 (M, SD) | Changes Across Practice Day |
| --- | --- | --- | --- |
| Success Rate | (%) | (%) |  |
| U-Shape | 9.83, 3.88 | 19.38, 5.65 | F(3.09,27.83)=8.85, p<0.001 *** |
| J-Shape | 12.79, 4.91 | 23.58, 5.47 | F(1.78,16.00)=14.15, p<0.001 *** |
| Box-Shape | 23.13, 8.44 | 49.79, 13.76 | F(3.10,27.86)=27.41, p<0.001 *** |
| I-Shape | 46.63, 23.90 | 67.58, 13.01 | F(2.38,21.40)=7.06, p=0.003 ** |
| Performance Error | (cm) | (cm) |  |
| U-Shape | 6.66, 2.59 | 3.20, 0.69 | F(1.30,11.66)=17.05, p=0.001 ** |
| J-Shape | 5.25, 2.10 | 2.48, 0.63 | F(1.90,17.05)=15.44, p<0.001 *** |
| Box-Shape | 2.95, 1.01 | 1.20, 0.47 | F(2.00,18.04)=29.86, p<0.001 *** |
| I-Shape | 1.76, 1.40 | 0.80, 0.16 | F(1.16,10.39)=4.75, p=0.049 * |
| Timing Error | (ms) | (ms) |  |
| U-Shape | 20.05, 3.24 | 16.20, 2.53 | F(2.67,24.03)=7.23, p=0.002 ** |
| J-Shape | 31.45, 8.75 | 23.00, 4.92 | F(2.04,18.39)=5.52, p=0.013 * |
| Box-Shape | 33.35, 13.64 | 24.65, 4.44 | F(2.43,21.83)=4.36, p=0.020 * |
| I-Shape | 30.20, 21.14 | 13.60, 4.54 | F(1.37,12.34)=6.20, p=0.021 * |
| Timing Window | (ms) | (ms) |  |
| U-Shape | 7.43, 2.09 | 12.88, 2.51 | F(2.29,20.58)=19.27, p<0.001 *** |
| J-Shape | 15.01, 4.43 | 23.69, 7.29 | F(2.79,25.12)=7.37, p=0.001 ** |
| Box-Shape | 29.22, 14.40 | 54.84, 20.78 | F(2.92,26.28)=13.97, p<0.001 *** |
| I-Shape | 43.21, 4.76 | 39.48, 7.99 | F(3.08,27.76)=1.825, p=0.165 |
