# Supplementary material for: Exploiting the geometry of the solution space to reduce sensitivity to neuromotor noise
Source: PLoS Comput Biol. 2018 Feb 20;14(2):e1006013. doi: 10.1371/journal.pcbi.1006013 (PMC5834204; doi:10.1371/journal.pcbi.1006013)
Supplement: S3 Text — Transformation from polar coodinatates to Cartesian coordinates of the workspace. (DOCX) [file pcbi.1006013.s003.docx]

**Supplement 3.**  **Invariance to** **Coordination Transformations**

The performance error in the skittles task was calculated from the states of the ball at the time of release, quantified by angular position and velocity of the lever arm at release. However, the physical model of the skittles task can be described in many different coordinates ([Sternad et al., 2010](#_ENREF_44)). An alternative reference frame to the polar coordinates with their origin at the pivot of the lever arm was given by the Cartesian coordinates of the workspace (Fig S3, panels A and B, and Fig 5A). The polar coordinates of the arm trajectory at ball release were transformed to Cartesian coordinates by the simple but nonlinear transformation:

$v_{x}=\dot{\theta}\sin\theta$ (S12)

$v_{y}=\dot{\theta}\cos\theta$ (S13)

which decomposed the angular position and velocity vector into velocity vector with its components in the *x*- and *y*-direction. The solution spaces of the same task in polar and Cartesian coordinates are shown in Fig S4, panels C and D, and Fig 5B. As can be seen, the solution space is rotated by approximately 90 degrees and the curvature of the result surface changed due to the nonlinear transformation. Analogous calculations of the performance and timing measures from the raw data confirmed that the dependent measures were invariant with respect to a change in coordinates.


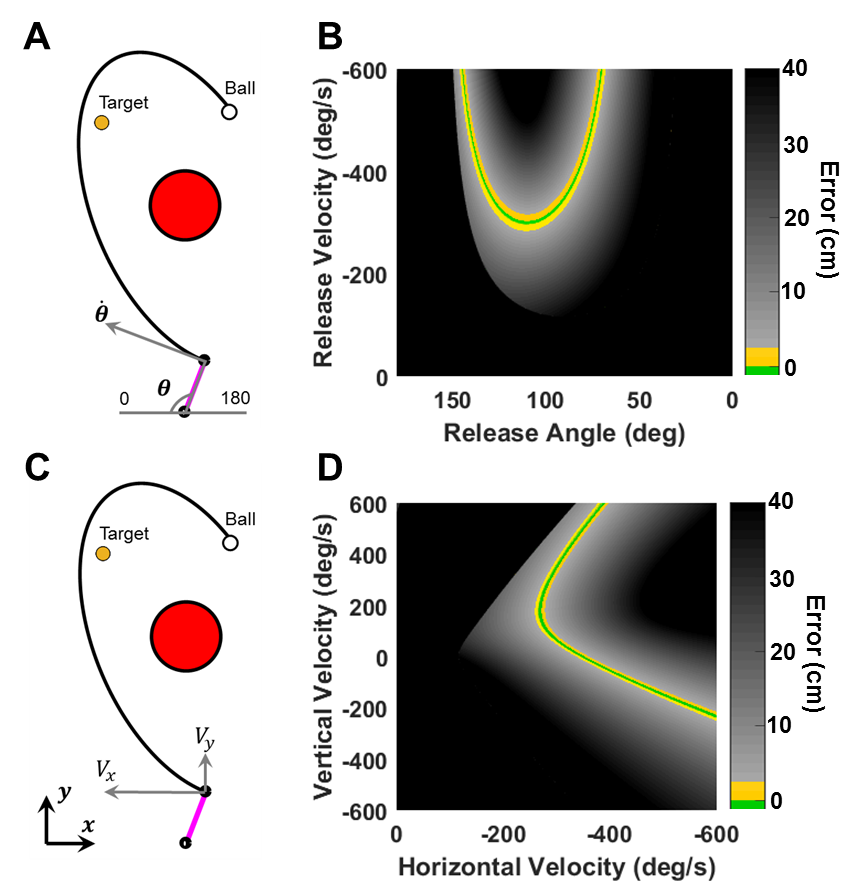


**Fig S3.** Solution space of the U-Shape task in two different coordinates. **A, B:** Workspace and solution space of the skittles task with polar coordinates. **C, D:** Workspace and solution space of the same task in Cartesian coordinates.
